# Differential functions of ERK1 and ERK2 in lung metastasis processes in triple-negative breast cancer

**DOI:** 10.1038/s41598-020-65250-3

**Published:** 2020-05-22

**Authors:** Maria Gagliardi, Mary Kathryn Pitner, Jihyun Park, Xuemei Xie, Hitomi Saso, Richard A. Larson, Rachel M. Sammons, Huiqin Chen, Caimiao Wei, Hiroko Masuda, Gaurav Chauhan, Kimie Kondo, Debu Tripathy, Naoto T. Ueno, Kevin N. Dalby, Bisrat G. Debeb, Chandra Bartholomeusz

**Affiliations:** 1 Section of Translational Breast Cancer Research, Department of Breast Medical Oncology, The University of Texas MD Anderson Cancer Center, Houston, Texas USA; 2 Department of Experimental Radiation Oncology, The University of Texas MD Anderson Cancer Center, Houston, Texas USA; 3 Division of Chemical Biology and Medicinal Chemistry, The University of Texas at Austin, College of Pharmacy, Austin, TX USA; 4 Department of Biostatistics, The University of Texas MD Anderson Cancer Center, Houston, Texas USA; 5 Present Address: Department of Breast Surgical Oncology, Advanced Cancer Translational Research Institute, Showa University, Tokyo, Japan

**Keywords:** Cancer, Cell biology, Oncology

## Abstract

Triple-negative breast cancer (TNBC) is an aggressive form of breast cancer characterized by metastasis, drug resistance and high rates of recurrence. With a lack or targeted therapies, TNBC is challenging to treat and carries a poor prognosis. Patients with TNBC tumors expressing high levels of ERK2 have a poorer prognosis than those with low ERK2-expressing tumors. The MAPK pathway is often found to be highly activated in TNBC, however the precise functions of the ERK isoforms (ERK1 and ERK2) in cancer progression have not been well defined. We hypothesized that ERK2, but not ERK1, promotes the cancer stem cell (CSC) phenotype and metastasis in TNBC. Stable knockdown clones of the ERK1 and ERK2 isoforms were generated in SUM149 and BT549 TNBC cells using shRNA lentiviral vectors. ERK2 knockdown significantly inhibited anchorage-independent colony formation and mammosphere formation, indicating compromised self-renewal capacity. This effect correlated with a reduction in migration and invasion. SCID-beige mice injected via the tail vein with ERK clones were employed to determine metastatic potential. SUM149 shERK2 cells had a significantly lower lung metastatic burden than control mice or mice injected with SUM149 shERK1 cells. The Affymetrix HGU133plus2 microarray platform was employed to identify gene expression changes in ERK isoform knockdown clones. Comparison of gene expression levels between SUM149 cells with ERK2 or ERK1 knockdown revealed differential and in some cases opposite effects on mRNA expression levels. Those changes associated with ERK2 knockdown predominantly altered regulation of CSCs and metastasis. Our findings indicate that ERK2 promotes metastasis and the CSC phenotype in TNBC.

## Introduction

Triple-negative breast cancer (TNBC), is an aggressive subtype that accounts for 10–15% of all breast cancers. As TNBC is characterized by the absence of HER2 and hormone receptor expression (estrogen and progesterone receptors), there are currently no US Food and Drug Administration-approved targeted therapies^1^. TNBC patients generally have a poor prognosis due to metastasis, high rates of recurrence and drug resistance.

Aberrant activity in the MAPK pathway (RAS-MEK-ERK) is important in the initiation and progression of cancer. Activation of this pathway is a marker of breast cancer metastasis and is clinically associated with shorter disease-free survival^2–5^. Recent work from our laboratory demonstrated that MEK inhibitors could reduce the cancer stem cell (CSC) population in TNBC, leading to a reduction of lung metastasis in a TNBC xenograft model^6^.

ERK, a member of the MAPK pathway, plays an essential role in cell proliferation and differentiation and facilitates cell migration through effects on cell-matrix contacts. ERK1 and ERK2 share 83% sequence identity^7^, are co-expressed in most tissues^7^, and are dually phosphorylated by MEK on threonine and tyrosine residues. While the two isoforms have many common substrates, it is unclear whether they also have unique substrates^8^. There is an ongoing debate as to whether ERK1 and ERK2 dictate functional differences or are functionally redundant; at the center of this debate is the question of whether global ERK function is determined by relative isoform expression levels *or* isoform specificity^9^.

We previously showed that TNBC patients with ERK2-overexpressing tumors had a poorer prognosis than TNBC patients with low-ERK2 expressing tumors^10^, suggesting that modulation of ERK2 could be a therapeutic strategy. Previous reports have shown that ERK2, but not ERK1, plays an essential role in the epithelial-mesenchymal transition (EMT), which is required for the acquisition of stem cell-like properties^11,12^. The transitional mesenchymal phenotype is a process required for metastasis involving loss of cell polarity, repression of epithelial genes, and an increase in motility and invasiveness^13,14^. In a pathologic context, these acquired characteristics enable cancer progression and metastasis. EMT is directly associated with the CSC phenotype in breast cancer, evidenced by an increased ability to form mammospheres^12^. TNBC is characterized by EMT and is highly associated with stem cell markers, which have been linked to biological aggressiveness^15^.

Here we provide evidence supporting the notion that ERK1 and ERK2 have functionally distinct properties and that ERK2, not ERK1, primarily contributes to lung metastasis in a TNBC mouse model. Gene expression microarray analysis of ERK1 knockdown vs. ERK2 knockdown revealed that genes with expression changes associated with ERK2 knockdown predominantly altered regulation of CSC and metastasis. Amongst these genes, EGR1 is an ideal candidate for further investigation, as its downstream targets affect cell growth, migration, and metastasis^16–19^. The knockdown of ERK2 resulted in significantly lower EGR1 at the mRNA level, validating our microarray data. Our findings indicate that ERK2 supports the CSC phenotype and metastasis in TNBC and reveal potential candidates (Table 1) for investigation in further mechanistic studies.

**Table 1:** Microarray identification of gene expression changes in Sum149 cells with ERK2 knockdown.

Gene Name	Fold Change shERK2 vs. shERK1	p value
*SOX7*	−9.065	0.000158473
*VIM*	−7.822	4.98E-06
*EGR1*	−6.123	0.000131334
*FN1*	−3.322	3.17E-05
*FZD3*	−2.655	0.000446418
*PARD6B*	−2.21	0.000116939
*BMPR1A*	−1.611	0.000656326
*WNT5A*	1.77	0.000383912

## Results

### ERK2 expression is associated with poor survival and is higher in TNBC than non-TNBC clinical samples

**Figure 1:**
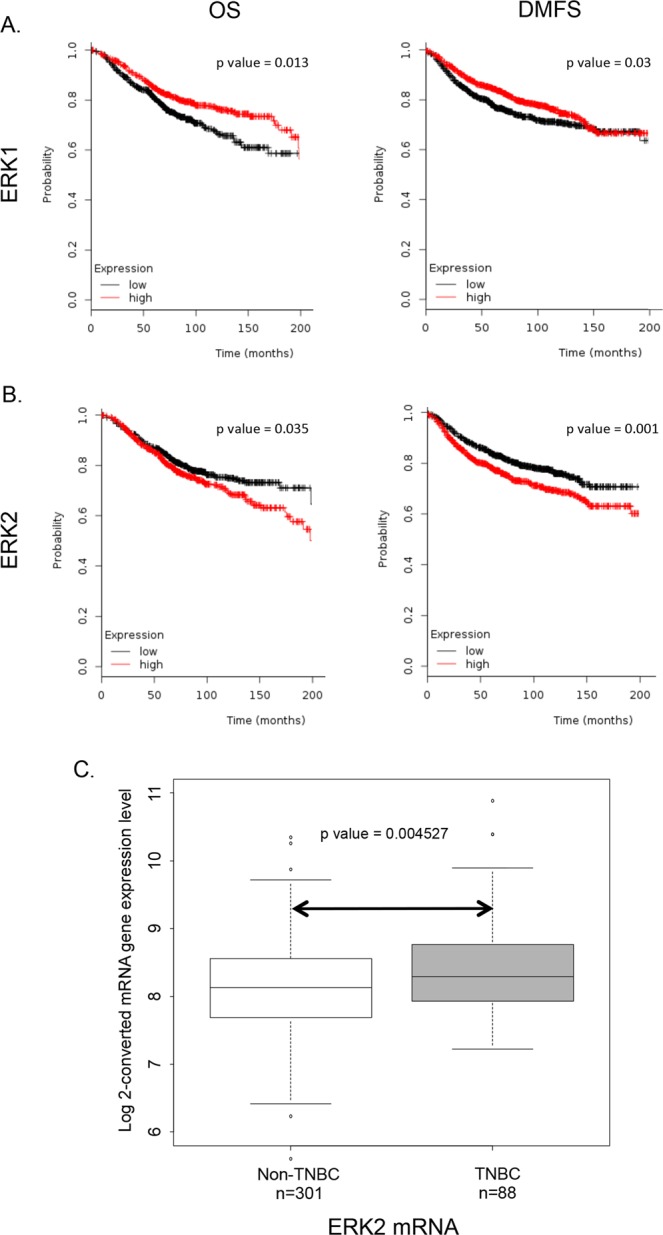
ERK2 expression is elevated in TNBC and is associated with poor overall survival (OS) and distant-metastasis-free survival (DMFS). (**A,B**) Kaplan-Meier plots of OS and DMFS of breast cancer patients after diagnosis with high levels of ERK1 (**A**) or ERK2 (**B**). (**C**) Analysis of the IBC World Consortium dataset shows significantly higher ERK2 levels in TNBC patients than in non-TNBC patients.

The Kaplan-Meier plotter was used to investigate the expression of ERK1 (MAPK3) and ERK2 (MAPK1) across breast cancer samples from all breast cancer patients (not stratified by disease subtype). We found that high ERK1 mRNA expression correlated with improved overall survival (OS) and distant-metastasis-free survival (DMFS) (Fig. 1A), whereas high ERK2 mRNA expression significantly correlated with poor OS and DMFS (Fig. 1B). These findings were in line with previous work from our laboratory, showing that patients with high-ERK2-expressing TNBC tumors had a higher risk of death than those with low-ERK2-expressing tumors^10^.

Further, we used the IBC World Consortium data set, which contains mRNA expression data on both inflammatory breast cancer and non-inflammatory breast cancer from 3 institutions^20^ (IBC, n = 137; non-IBC, n = 252), together with Affymetrix gene chips, normalized with MAS5 algorithm, to analyze ERK2 (212271_at) mRNA gene expression stratified by estrogen receptor, progesterone receptor, and HER2 status. The TNBC subgroup showed higher ERK2 expression than the hormone receptor-positive, HER2-negative subgroup did (p < 0.001) (data not shown). When we compared TNBC vs. non-TNBC, the TNBC subgroup showed higher ERK2 expression (p = 0.004527). In summary, we found that TNBC patients had significantly higher ERK2 expression than non-TNBC patients (Fig. 1C), suggesting a role for ERK2, but not ERK1, in TNBC.

### Neither knockdown of ERK1 nor ERK2 has an effect on proliferation, but the loss of ERK2 inhibits anchorage-independent growth in TNBC cells

**Figure 2:**
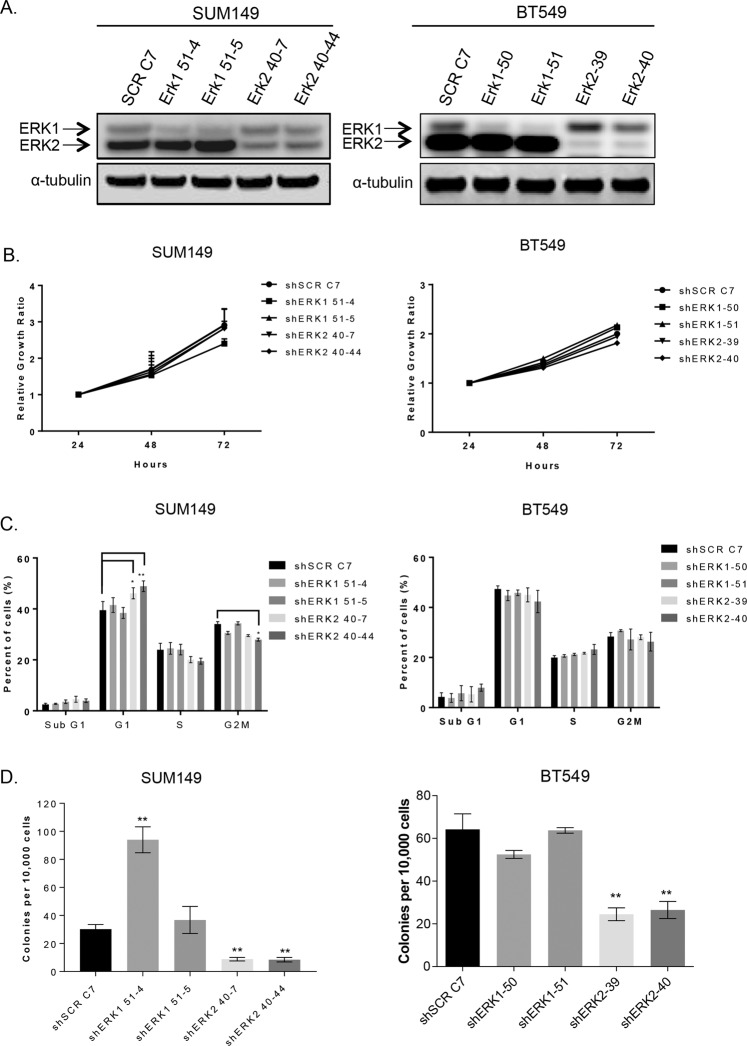
Stable ERK1/2 knockdown in TNBC cells has no effect on proliferation, but loss of ERK2 inhibits anchorage-independent growth in TNBC cells. (**A**) SUM149 and BT549 cells were used to create stable ERK1 knockdown cells (SUM149: ERK1 51-4, ERK1 51-5; BT549: ERK1-50, ERK1-51) and stable ERK2 knockdown cells (SUM149: ERK2 40-7, ERK2 40-44; BT549: ERK2-39, ERK2-40). Efficient knockdown compared to control scrambled shRNA (SCR)-expressing cells, was confirmed by immunoblot. (**B,C**) Knockdown of ERK1 or ERK2 in SUM149 or BT549 cells had no significant effect on cell proliferation assessed using CellTiter Blue (**B**) or cell cycle distribution analyzed using flow cytometry (**C**) Error bars represent the mean (n = 3) ± S.D. (**D**) Anchorage-independent growth in soft agar was reduced in ERK2 knockdown SUM149 and BT549 cells. The average of 2 independent experiments is shown. Error bars represent the mean (n = 3) ± S.E.M. **P < 0.001. Graphs were generated using GraphPad.

To determine the roles of the ERK1 and ERK2 isoforms, we created stable knockdown clones in SUM149 and pools in BT549 TNBC cells using shRNA lentiviral vectors. The knockdown of each ERK isoform was confirmed via western blot (Fig. 2A). No phenotypic changes were observed in either of the ERK isoform knockdowns. Using the Sox-Sub-D-based peptide sensor to detect changes in kinase activity in cell lysates^21^, we found lower total ERK activity in ERK1 and ERK2 knockdown cells than in control cells (Additional file 1: Supplementary Fig. 1A). These results suggest that knockdown reduces both gene expression and kinase activity. The fluorescent signal generated from the Sox-Sub-D peptide phosphorylation was measured, and lysates were tested for ERK activity in the ERK1 and ERK2 knockdown cells. ERK2 knockdown had a greater effect on overall ERK activity than ERK1 knockdown (Additional file 1: Supplementary Fig. 1B,C).

Next, we assessed cell proliferation in SUM149 and BT549 cells over 72 h using CellTiter Blue. The knockdown of ERK1 or ERK2 had no effect on cell proliferation (Fig. 2B). We also examined the effect of ERK isoform knockdown on cell cycle distribution. As shown in Fig. 2C, in both SUM149 and BT549 TNBC cells, knockdown of ERK1 or ERK2 did not significantly alter the cell cycle distribution. Anchorage-independent growth in soft agar, an *in vitro* marker of tumorigenicity was reduced by 74% and 60% with a loss of ERK2, but not ERK1, in both SUM149 and BT549 TNBC cells respectively (Fig. 2D).

### ERK2 is a potent driver of self-renewal capacity in TNBC

**Figure 3:**
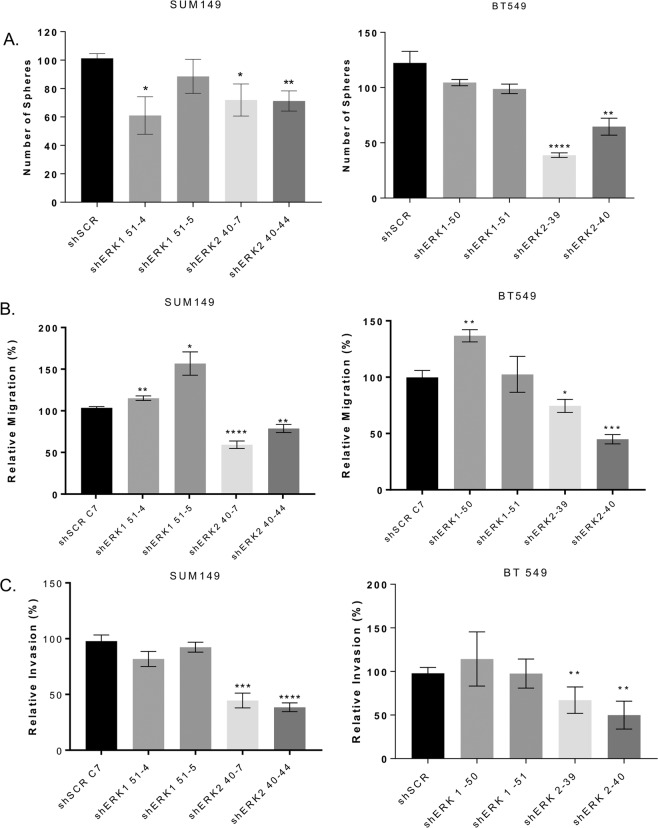
ERK2 knockdown reduces migration, invasion and mammosphere formation. (**A**) Mammosphere formation assay. ERK2 knockdown attenuated sphere formation in SUM149 (by 30%) and BT549 cells (by at least 48%). Migration (**B**) and invasion (**C**) assays were performed using transwell chambers. (**B**) ERK2 knockdown reduced migration in both SUM149 and BT549 cells by at least 22% and 26% respectively. (**C**) Invasion decreased in ERK2 knockdown cells by at least 56% and 33% in SUM149 and BT549 cells respectively. ERK1 knockdown increased migration but had no significant impact on invasion. Graphs were generated using GraphPad. Error bars represent the mean (n = 3) ± S.E.M. *P < 0.01; **P < 0.001; ***P = 0.0001; ****P < 0.0001; Student’s t-test.

Studies have shown that EMT leads to the generation of breast cancer cells with stem cell-like properties capable of self-renewal^12^. These cells can be enriched *in vitro* by growing them as mammospheres, which are 3-dimensional spherical breast cancer cell colonies that grow in suspension in serum-free, growth-factor-enriched media and are characterized by the expression of specific cell surface markers, such as CD44^+^/CD24^−/low^. To determine the impact of ERK1 and ERK2 on the self-renewal capacity of TNBC cells, we examined the effects of ERK1 or ERK2 knockdown on mammosphere formation and expression of CD44 and CD24 on the cell surface. The knockdown of ERK2 significantly decreased the formation of mammospheres in both SUM149 (by 30%, p = 0.01) and BT549 (by at least 48%, p = 0.0001) TNBC cell lines (Fig. 3A). Mammosphere formation is unaffected by the knockdown of ERK1 in BT549 cells, and only one ERK1 knockdown clone (shERK1 51-4) in SUM149 cells has decreased mammosphere formation (by 40%, p = 0.01) (Fig. 3A). Loss of ERK2 also decreased the fraction of SUM149 cells with CD44^+^/CD24^−/low^ surface marker expression pattern (Additional file 2: Supplementary Fig. 2); compared to the fraction of control cells, proportions of shERK2 40-7 and shERK2 40-44 CD44^+^/CD24^−/low^ cells were reduced by 80% and 70%, respectively. The knockdown of ERK1 in SUM149 cells has no effect on CD44^+^/CD24^−/low^ surface marker expression. These results suggested that ERK2 is a more potent driver than ERK1 of the self-renewal capacity of TNBC cells.

### Knockdown of ERK2 inhibits, whereas loss of ERK1 drastically increases, the metastatic burden in a TNBC model

We next investigated the role of ERK isoforms in the invasive phenotype of TNBC cells. In SUM149 and BT549 cells, compared to the migration of control cells, migration of ERK2 knockdown cells was reduced by at least 22% (*P* < 0.001) and 26% (*P* < 0.01), respectively (Fig. 3B). Similarly, in both SUM149 and BT549 cells, compared to invasion of control cells, invasion of ERK2 knockdown cells was reduced by at least 56% (*P* = 0.0001) and 33% (*P* < 0.002), respectively (Fig. 3C). Conversely, knockdown of ERK1 increased migration in both SUM149 and BT549 cells to varying degrees.

**Figure 4:**
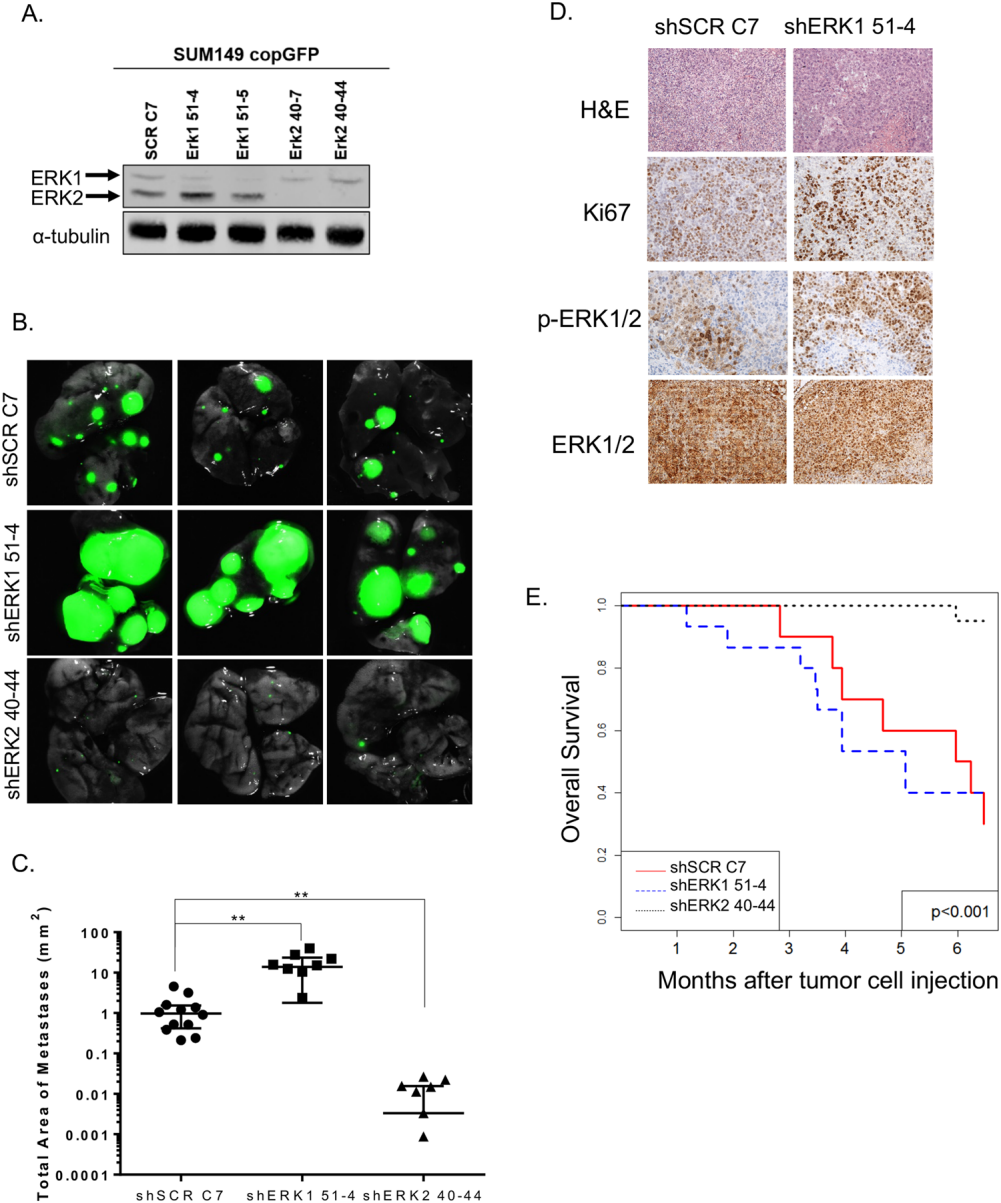
ERK isoforms have opposite effects on lung metastatic burden. (**A**) copGFP-labeled SUM149 SCR, shERK1, and shERK2 cells were generated, and ERK isoform knockdown was confirmed by immunoblotting. (**B,C**) Nine weeks after cancer cell injection, mice were sacrificed, lung metastatic burden was observed using stereomicroscopy, and total tumor area was quantified. ERK1 knockdown dramatically increased metastatic burden. ERK2 knockdown almost entirely prevented lung metastasis. (**D**) Micrographs (20X) of lung SUM149 SCR and SUM149 shERK1 tumors immunohistochemically stained for hematoxylin and eosin, proliferation marker Ki67, ERK, and phosphorylated ERK. (**E**) Kaplan-Meier survival analysis shows that mice bearing SUM149 shERK2 tumors survived longer than control mice or mice bearing SUM149 shERK1 tumors. No tumor tissue was identified in the lungs of mice injected with shERK2 40-44 cells.

Based on our findings that ERK2 is a more potent driver of the CSC phenotype and invasiveness than ERK1, as demonstrated by decreased mammosphere formation, migration, and invasion (Fig. 3), we hypothesized that knockdown of ERK2 would prevent metastasis of TNBC cells in an animal model. We labeled SUM149 SCR, shERK1, and shERK2 cells with a green fluorescent protein (copGFP) and sorted the cells via FACS analysis; we then confirmed ERK isoform knockdown by immunoblotting (Fig. 4A). SUM149 SCR C7, shERK1, and shERK2 copGFP-labeled cells were injected into the tail vein of female SCID-beige mice. Animals were weighed biweekly, and no significant difference in body weight was seen between the different groups throughout the experiment (Additional File 3: Supplementary Fig. 3A). Animals were sacrificed at 9 weeks. Compared with the metastatic burden in mice injected with SUM149 SCR C7 cells, the metastatic burden in mice injected with SUM149 shERK2 cells was significantly lower. In contrast, the metastatic burden in mice injected with SUM149 shERK1 cells was considerably higher, as revealed by GFP signal via stereomicroscopy (Fig. 4B) and quantified by measurement of the total area of metastasis (Fig. 4C). To assess the impact of ERK1 and ERK2 knockdown on metastasis, we collected the lung tissue from the mice, stained the tissue with hematoxylin-eosin, and analyzed metastatic lesions and proliferation (Ki-67) (Fig. 4D). However, the minimal size and the number of metastatic lesions in the mice injected with SUM149 shERK2 cells precluded any identification of metastasis in those mice (Additional file 3: Supplementary Fig. 3B).

Given the significant difference in metastatic burden between mice injected with SUM149 SCR C7, shERK1, and shERK2 mice, a separate survival experiment was performed. The analysis showed that mice injected with shERK2 cells had significantly better OS than mice injected with SCR C7 cells or shERK1 cells (Fig. 4E, Additional file 4: Supplementary Table 1), complementing the clinical data presented in Fig. 1, which indicated that ERK2 plays a significant role in patient OS.

### Global gene expression changes associated with ERK2 knockdown predominantly alter the regulation of CSC and metastasis

**Figure 5:**
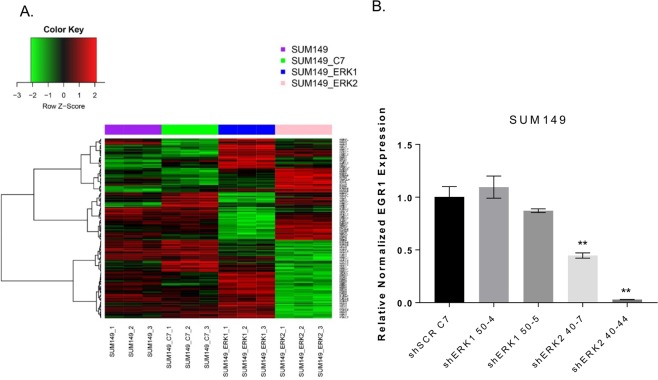
EMT gene expression decreases upon ERK2 knockdown. (**A**) The Affymetrix HGU133plus2 microarray platform was used to compare gene expression changes in SUM149 SCR C7, shERK1 51-4, and shERK2 40-44 cell lines. Using a FDR of 0.01, a heatmap of 149 genes was generated, revealing reduced expression of EMT-associated genes in ERK2 knockdown cell lines. (**B**) EGR1 expression was significantly reduced in SUM149 shERK2 40-7 (by 66%) and shERK2 40-44 cells (by 97%). **P < 0.001 Graphs were generated using GraphPad. Error bars represent the mean (n = 3) ± S.D.

The Affymetrix HGU133plus2 microarray platform was used to compare gene expression changes in SUM149 SCR C7, shERK1 51-4, and shERK2 40-44 cell lines. There were 3, 50, 147, 1306, and 3539 genes with a significantly different expression between at least 2 groups at false discovery rates (FDRs) of 0.001, 0.005, 0.01, 0.05, and 0.1, respectively (Additional file 5: Microarray results, Additional file 6. Supplementary Table 2). We selected an FDR of 0.01 and generated the heatmap of gene expression using the 147 selected genes for the overall F test across all groups (Fig. 5A). These global gene expression changes suggested evidence of a functional difference between ERK1 and ERK2, with some transcription factors whose expression levels changed in opposite directions upon ERK1 or ERK2 knockdown. These transcription factors will be explored further in future studies.

The 147 genes depicted in the heatmap at FDR of 0.01 were imported into Ingenuity Pathway Analysis software (www.ingenuity.com) for further analysis of the complex relationships between ERK2 and the microarray targets. Global gene expression changes associated with knockdown of ERK2 were seen in the regulation of metastasis (Table 1). Among the genes with ERK2-knockdown-associated expression change was *EGR1*, which was down-regulated 6-fold (p = 0.00013) compared to shERK1 cells as confirmed by qPCR (Table 1, Fig. 5B, Additional file 3: Supplementary Fig. 3C). In our future studies, we will pursue EGR1, an attractive mechanistic candidate to pursue as it is an immediate-early response transcription factor that is stimulated by growth factors, cytokines, and stress and impacts cell growth, migration, angiogenesis, and metastasis^19,22–24^. Furthermore, EGR1 expression is mediated through the MAPK pathway making it a relevant candidate to pursue in the mechanistic studies^25^.

## Discussion

Our findings demonstrated that ERK2, but not ERK1, promotes migration and invasion, the CSC phenotype, and metastasis in TNBC. Furthermore, our findings suggest that ERK2 may exert its impact on TNBC in part through EGR1.

The MAPK pathway acts by transferring growth-promoting signals from the cell surface or cytoplasm to the nucleus through a kinase cascade. Aberrant activity in this pathway is important in the initiation and progression of many of the hallmarks of cancer. Mutations in *RAS and RAF* are frequent events in many carcinomas, including lung, colorectal, pancreatic, and melanoma^26–30^.

While the frequency of RAS/MAPK activating mutations in breast cancers is low, the pathway is hyperactive in almost half of breast cancers^31^. There are transcriptional signatures of activated MAPK in TNBC and basal-like breast cancers, suggesting significant oncogenic activity. Therapeutic targeting of the RAS/MAPK pathway with specific small molecule inhibitors of RAF and MEK has been effective in the treatment of advanced melanomas. Mounting preclinical evidence supports targeting the RAS/MAPK cell signaling pathway in the TNBC subtype, despite extensive genomic surveys such as The Cancer Genome Atlas demonstrating rare canonical mutations in this pathway. Lack of somatic mutations in the pathway does not imply that breast cancers, specifically TNBC, do not utilize this pathway.

Numerous studies have focused on determining whether differential roles or functional redundancy characterize the ERK isoforms^9,32,33^. In our present study, knocking down endogenous ERK2 had a generally greater effect than knocking down endogenous ERK1;we show that ERK2 is critically important in driving *in vitro* colony formation, migration and invasion, and the CSC phenotype in TNBC. However, the possibility that ERK1 may contribute to these cellular phenotypes cannot be dismissed. There was more ERK2 than ERK1 in both cell lines that we studied, and therefore it could also be concluded that the phenotypes associated with ERK2 knockdown also correspond to the more significant knockdown of ERK activity. Nonetheless, we showed striking functional differences in an experimental metastasis model, supporting our hypothesis that ERK1 and ERK2 have differential functions *in vivo*. Mice injected with TNBC cells with ERK2 knocked down had significantly lower lung metastatic burden than mice injected with control or shERK1 cells and had significantly improved OS compared to the shERK1 group. Taken together, the data suggests that ERK2 is a more potent driver of the metastatic phenotype than ERK1, which seems to have a metastasis-suppressing role. Again, these results support the independent functions of the ERK isoforms^34–36^. The role of ERK1 in this surprising phenotype will be explored in future studies as the scope of this work was focused more on reducing phenotypes associated with malignancy.

Microarray analysis of shERK2 versus shERK1 cells further attributed different functions to the ERK isoforms. Decreased expression of CSC-associated and metastasis-associated genes was found in ERK2 knockdown but not ERK1 knockdown cells. Among these genes, *EGR1* exhibited a significant decrease in shERK2 cells. While EGR1 has been described as having both positive and negative functions in tumor development^23,37–40^, our data suggest that in TNBC, EGR1 promotes tumor progression under the control of ERK2. Previously, ERK-ERF-EGR1 was reported to be a novel switch inducing mammary cell migration^16,17^, and inhibition of EGR1 expression can reverse the transformation of prostate cancer cells *in vitro* and *in vivo*^41^. EGR1 has also been shown to contribute to the maintenance and proliferation of stem-like cells in glioblastoma^42^. We validated EGR1 as a target of interest as knockdown of ERK2, but not ERK1, which resulted in significantly lower *EGR1* mRNA levels.

Previous work has identified elevated EGR1 expression as a marker for drug resistance in non-TNBC (in MCF-7 cells, which are ER+)^43^; our results indicate that EGR1 could be a therapeutic biomarker in TNBC. It has already been identified as such in other cancers (hepatocellular carcinoma and gastric cancer^44,45^). To develop EGR1 as a relevant target in TNBC patients, we first need to better understand its role in metastasis and CSC phenotype in future studies. Insight into the biology of the ERK2-EGR1 axis and the mechanism by which it acts to promote metastasis and CSC phenotype is highly relevant to improving the outcome of TNBC patients.

The best patient population for ERK inhibition has not yet been identified. There is interest in the combined use of inhibitors targeting different components of the same pathway; this could be prudent in the case of MAPK pathway targeting as resistance to RAF and MEK inhibitors frequently involves the recovery of ERK signaling. This raises the question: could a specific ERK isotype inhibitor increase the potency and durability of MAPK pathway inhibition?

## Conclusions

Our findings support the idea that, unlike ERK1, ERK2 promotes metastasis and the CSC phenotype in TNBC. This was determined using stable knockdown clones of ERK1 and ERK2 in SUM149 and BT549 TNBC cells using shRNA lentiviral vectors both *in vitro* and *in vivo*. The work shown here lays the groundwork for future studies to explore the ERK2 EGR1 axis and potential therapeutic applications. EGR1 may be a relevant therapeutic target in treating this highly aggressive breast cancer subtype.

## Methods

### Cell lines and cell cultures

SUM149 cells were purchased from Asterand (Detroit, MI) and cultured in F12 medium (Sigma) supplemented with fetal bovine serum (FBS; 5%), penicillin-streptomycin (100 units/mL), insulin (5 μg/mL), and hydrocortisone (1 μg/mL). BT549 cells were purchased from ATCC (Manassas, VA) and cultured in RPMI-1640 medium (Sigma) supplemented with FBS (10%) and penicillin-streptomycin (100 units/mL). Cells were passaged every 3 days and authenticated twice a year at the Characterized Cell Line Core Facility at MD Anderson Cancer Center through genotyping (in August 2014, October 2014, January 2015, November 2018). Stable cell lines were created using an shRNA lentiviral system (Mission shRNA Lentiviral System, Sigma). Briefly, SUM149 and BT549 cells were transduced with shRNA against scrambled control, ERK1 (TRCN0000006150: CCGGCCTGAATTGTATCATCAACATCTCGAGATGTTGATGATACAATTCAGGTTTTT; TRCN0000006151: CCGGCGACCTTAAGATTTGTGATTTCTCGAGAAATCACAAATCTTAAGGTCGTTTTT), and ERK2 (TRCN0000010039: CCGGTGGAATTGGATGACTTGCCTACTCGAGTAGGCAAGTCATCCAATTCCATTTTT; TRCN0000010040: CCGGCAAAGTTCGAGTAGCTATCAACTCGAGTTGATAGCTACTCGAACTTTGTTTTT); stably transfected cells were selected in media containing puromycin. Stable SUM149 copGFP-labeled cells were created by infection with the pCMV-copGFP lentiviral vector (System Biosciences) and sorted by flow cytometry-based on GFP expression.

### Western blot analysis

Collection of cell proteins and western blot analysis were performed as previously described^6^. Primary antibodies used were anti-p44/42 MAPK (Erk1/2) (1:1 000 dilution Cell Signaling), anti-phospho-p42/44 MAPK (Thr202/Tyr204) (1:1 000 dilution; Cell Signaling), anti-α-tubulin (1:5 000 dilution; Sigma-Aldrich) and anti-EGR1 (1:1 000 dilution; Cell Signaling). Secondary antibodies were horseradish peroxidase-conjugated IgG (1:10 000 dilution; Invitrogen) for chemiluminescent signal detection and the corresponding Alexa Fluor-conjugated IgG (1:5 000 dilution; Invitrogen) for fluorescence signal detection.

### Proliferation assay

To assess the effect of ERK isoform knockdown on cell proliferation, CellTiter Blue (Promega) was used, as previously described (17). SUM149 and BT549 cells (2 × 10^3^/100 µL) were seeded into a 96-well plate, and measurements were made at 24, 48, and 72 h.

### Cell cycle distribution analysis

As described previously^6^, flow cytometry was used to determine the cell cycle distribution of SUM149 and BT549 cells (2 × 10^5^), which were plated in 6-well plates and cultured for 72 h. Cells were then treated with RNaseA (32 mg/ml) and stained with propidium iodide (1 mg/ml).

### ERK sensor assay: fluorescence assay

The original protocol for the quantification of ERK activity in cell lysates was described by Warthaka *et al*.^21^. Here, 20 µg protein from cell lysate was added to assay buffer (25 mM HEPES, pH 7.6, 50 mM KCl, 0.1 mM EDTA, 0.1 mM EGTA, 2 mM DTT, 10 mM MgCl_2_, 10 µg/mL BSA) with or without the ERK inhibitor SCH772984 (50 nM, final DMSO concentration 2% v/v) to detect background phosphorylation of the fluorescent peptide. Reactions were initiated by the addition of 0.5 mM MgATP and 20 µM fluorescent peptide Sox-Sub-D. The reactions were performed at 26 °C in 60-µL volumes and read by using a Synergy H4 plate reader (BioTek, Winooski, VT, USA), with fluorescence measurements (λex = 360 nm; λem = 482 nm) every 10 s for a total of 20 min. Reaction rates were measured from the slope of each data set. Rates were expressed in units of concentration/time by using a conversion factor, given that the relationship between the Sox-Sub-D fluorescence signal and its concentration is linear. The conversion factor was obtained by measuring the maximum fluorescence signal for complete phosphorylation of 20 µM Sox-Sub-D.

### ERK sensor assay: western blot analysis

Western blots of the lysates were also performed to evaluate relative ERK activity. 30–40 micrograms of protein from each lysate were separated on a 10% SDS-polyacrylamide gel (BioRad, Hercules, CA, USA) and transferred onto Immobilon-FL PVDF membrane (Millipore, Burlington, MA, USA). Primary antibodies were incubated with the membranes overnight at 4 °C according to the following dilutions: 1: 2 000 anti-phospho-p42/44 MAPK (Erk1/2) (Thr202/Tyr204) (E10) mouse monoclonal antibody (Cell Signaling Technology); 1:1 000 anti-p44/42 MAPK (Erk1/2) (137F5) rabbit monoclonal antibody (Cell Signaling Technology); and 1:50 000 anti-alpha-Tubulin (EP1332Y) rabbit monoclonal antibody (Millipore). The membranes were incubated with secondary antibodies at room temperature for 1 h (1:15 000 IRDye 800 CW goat (polyclonal) anti-rabbit IgG or IRDye 680RD goat (polyclonal) anti-mouse IgG (LI-COR)). Membrane fluorescence was detected on an Odyssey imaging system (LI-COR, Lincoln, NE, USA).

### Soft agar assay

As described previously^6^, a bottom agarose layer (1%) was laid in 12-or 6-well plates. Cells (1 × 10^4^ cells/well), resuspended in 2 mL of 0.5% agarose solution in complete medium, were overlaid and incubated for 25 days. Colonies formed were stained using MTT (3-(4,5-dimethylthiazol-2-yl)-2,5-diphenyltetrazolium bromide) (Sigma), and those greater than 80 μm in diameter were counted using the GelCount system (Oxford Optronix, UK) according to the manufacturer’s instructions.

### Migration and invasion assay

As described previously^6^, migration assays were performed in triplicate using a 24-well micro-chemotaxis chamber. Invasion assays were performed using 24-well Growth Factor Reduced Corning Matrigel Invasion Chamber (Corning, NY, USA). For both assays, SUM149 and BT549 cells (1 × 10^5^/350 μL) were resuspended in FBS-free medium and added into appropriate chambers. The bottom chamber was filled with complete medium (750 μL) containing 10% FBS as an attractant. The cells were allowed to migrate for 6 h (migration) or 24 h (invasion) and were then fixed and stained with hematoxylin and eosin. Migrated and invaded cells were scanned using the PathScan Enabler IV Histology Slide Scanner (Meyer Instruments, Inc., TX, USA) and quantified using National Institutes of Health Image J software (http://rsb.info.nih.gov/ij/).

### Mammosphere formation assay

As described previously^6^, mammosphere formation assay was performed as previously described^6^. Single-cell suspensions of SUM149 cells (2 × 10^4^ cells/well) and BT549 cells (1 × 10^4^ cells/well), in MammoCult Human Medium Kit (StemCell Technologies, Vancouver, Canada), were seeded in 6-well ultra-low attachment plates (Corning Incorporated Costar, Corning, NY, USA). After a 7-day incubation, mammospheres were stained using MTT (3-(4,5-dimethylthiazol-2-yl)-2,5-diphenyltetrazolium bromide) (Sigma), and spheres greater than 80 μm in diameter were counted using the GelCount system (Oxford Optronix, UK) according to the manufacturer’s instructions.

### CSC subpopulation analysis

As described previously^6^, SUM149 and BT549 cells (3 ×10^5^ cells) were seeded in 60-mm plates, and 48 h later, cells were harvested and incubated at 37 °C with ALDEFLUOR reagent (STEMCELL Technologies Inc.) or at room temperature with anti-CD24 and anti-CD44 antibodies (BD Biosciences) for 30 min. Samples were analyzed by flow cytometry. As previously described, specific controls were used for each subpopulation analyzed. CD24−/CD44+ subpopulation: cells incubated with CD24-PE alone or CD44-FITC alone to determine nonspecific signals and to gain the gates for CD24+ and CD44+ subpopulations, respectively.

### *In vivo* experimental metastasis model

The Institutional Animal Care and Use Committee (The University of Texas MD Anderson Cancer Center) approved this study and all experiments were performed in accordance with relevant guidelines and regulations. Female SCID-beige mice, 6–8 weeks of age, average weight 20 g, were randomly divided into 3 groups of 15 mice each. Suspensions of SUM149 copGFP-labeled cells (1 × 10^6^ cells in 0.2 ml of PBS) were injected via a tail vein under aseptic conditions. Mice were weighed biweekly for 9 weeks. At 9 weeks, whole lungs were collected for analysis of copGFP signal intensity under a stereomicroscope. Samples were processed for immunohistochemistry staining.

### *In vivo* survival assay

The Institutional Animal Care and Use Committee (The University of Texas MD Anderson Cancer Center) approved this study and all experiments were performed in accordance with relevant guidelines and regulations. Female SCID-beige mice, 6–8 weeks of age, average weight 20 g, were randomly divided into 5 groups of 10 mice each. Suspensions of SUM149 copGFP-labeled cells (1 × 10^6^ cells in 0.2 mL of PBS) were injected via a tail vein under aseptic conditions. Mice were weighed biweekly until moribund, at which point they were euthanized. The power of this study was calculated based on the assumption that the hazard rates are proportional, using 2-sided log-rank test between the ERK1 and ERK2 groups at a 0.05 significance level to detect a difference of 0.5500, the difference between the proportion surviving in the ERK1 group (0.4000) and the proportion surviving in the ERK2 group (0.9500) (NCSS-PASS, 2005). The distribution of OS by the animal group was evaluated by the Kaplan and Meier method, and the groups were compared using the log-rank test.

### Immunohistochemistry staining

As previously described^6^, tumor tissues were fixed in formalin, embedded in paraffin, sectioned to 5 µm, and mounted on slides. The sections were deparaffinized in xylene, rehydrated in graded alcohols, and washed in distilled water. Antigens on sections were retrieved by boiling in 10 mM citric acid (pH 6.0) for 40 min. Endogenous peroxidases were quenched by incubation in 3% H_2_O_2_ for 10 min at room temperature. The slides were washed 3 times with PBS and blocked for 30 min with 10% normal goat serum in 1% bovine serum albumin/PBS. The slides were then incubated with the following antibodies: anti-Ki-67 (Lab Vision), anti-phospho-p42/44 MAPK (Thr202/Tyr204) (Cell Signaling) and anti-p44/42 MAPK (Erk1/2) (Cell Signaling). Stained slides were visualized and acquired with an Eclipse 80i microscope (Nikon) at 20x magnification.

### Microarray analysis

SUM149 parental, SCR C7, shERK1 51-4, and shERK2 40-44 cell lines were compared in triplicate on the Affymetrix HGU133plus2 microarray platform (Affymetrix, CA, USA). We used the Robust Multiarray Analysis algorithm, which borrows strength across arrays, to normalize and quantify the data. We performed a feature-by-feature analysis of variance (ANOVA) with multiple contrasts of interest between cell line groups. Multiple contrasts were performed using the multcomp package. We used beta-uniform mixture models to fit the resulting p values of the overall ANOVA models in order to adjust for multiple testing. We computed the cutoff p values and the number of significant genes using several different FDRs. To manifest the differences in different cell lines, a heatmap was plotted using the selected genes at an FDR of 0.01 across all cell lines. Spearman correlation coefficient was applied to compute the distance between the samples. Ward’s linkage was used as the clustering method. The statistical analyses were performed in R.

### Quantitative real-time PCR

Total RNA was isolated from the SCR C7, shERK1 51-4, and shERK2 40-44 cells (Invitrogen), and 1 μg of total RNA was reverse-transcribed to generate the first strand of cDNA using random hexamer primer and reverse transcriptase (Invitrogen). SYBR Green-based (Bio-Rad) real-time PCR was carried out using the following primers for EGR1 and GAPDH: EGR1 forward: CTTCAACCCTCAGGCGGACA; reverse: GGAAAAGCGGCCAGTATAGGT; GAPDH forward: ACCCAGAAGACTGTGGATGG; reverse: TCTAGACGGCAGGTCAGGTC.

### Kaplan meier plots

Kaplan Meier plotter is an online (https://kmplot.com/analysis/) tool developed to identify subsets of genes/mRNAs that are associated with disease progression in breast cancer, among others. It analyses the effect of 54 thousand genes on cancer prognosis. Breast cancer dataset includes 6,234 samples.

### Statistical analysis

Statistical analyses of *in vitro* studies were performed with Prism, version 5 (GraphPad Software, Inc). Data are presented as means ± standard error or standard deviations. Means for all data were compared by 1-way ANOVA with post hoc testing or by unpaired t-test. p < 0.05 was considered statistically significant.

## Supplementary information

Supplementary Information
