# Supplementary material for: Differential functions of ERK1 and ERK2 in lung metastasis processes in triple-negative breast cancer
Source: Sci Rep. 2020 May 22;10:8537. doi: 10.1038/s41598-020-65250-3 (PMC7244517; doi:10.1038/s41598-020-65250-3)

Supplemental Figures and Tables

Differential functions of ERK1 and ERK2 in lung metastasis processes in triple-negative breast cancer

Maria Gagliardi[1*], Mary Kathryn Pitner[1*], Jihyun Park[1], Xuemei Xie[1], Hitomi Saso[1], Richard A. Larson[2], Rachel M. Sammons[3], Huiqin Chen[4], Caimiao Wei[4], Hiroko Masuda[1§], Gaurav Chauhan[1±], Kimie Kondo[1], Debu Tripathy[1], Naoto T. Ueno[1], Kevin N. Dalby[3], Bisrat G. Debeb[1], and Chandra Bartholomeusz[1]

[1]Section of Translational Breast Cancer Research, Department of Breast Medical Oncology, The University of Texas MD Anderson Cancer Center, Houston, Texas, USA

[2]Department of Experimental Radiation Oncology, The University of Texas MD Anderson Cancer Center, Houston, Texas, USA

[3]Division of Chemical Biology and Medicinal Chemistry, The University of Texas at Austin, College of Pharmacy, Austin, TX, USA

[4]Department of Biostatistics, The University of Texas MD Anderson Cancer Center, Houston, Texas, USA

*These authors contributed equally to this work.

Current address:

§ Department of Breast Surgical Oncology, Advanced Cancer Translational Research Institute, Showa University, Tokyo, Japan

Corresponding author and requests for reprints: Chandra Bartholomeusz, Department of Breast Medical Oncology, Unit 1354, The University of Texas MD Anderson Cancer Center, 1515 Holcombe Boulevard, Houston, TX 77030. Tel: 713-745-1086; E-mail: chbartho@mdanderson.org

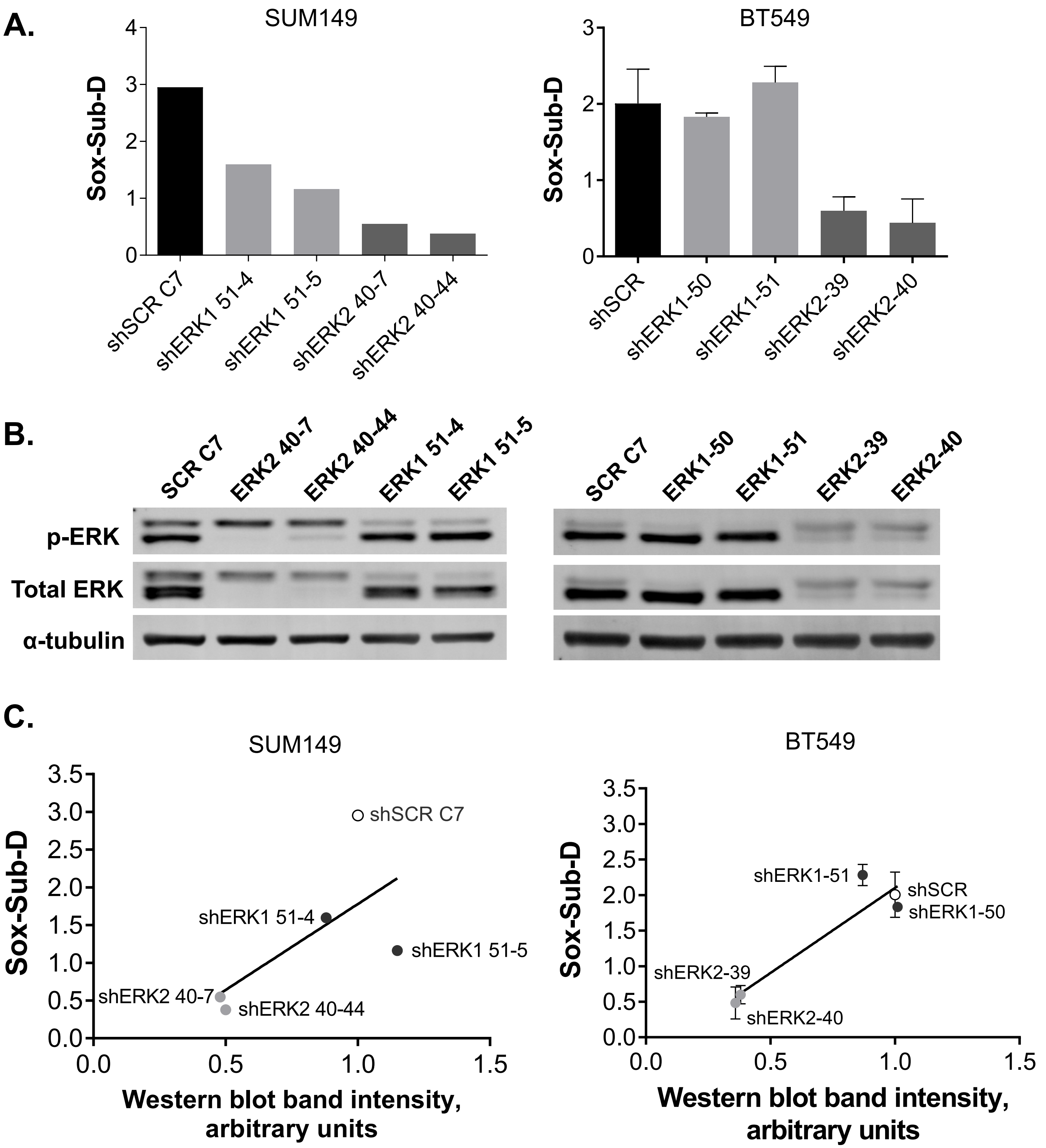

Supplementary Fig.1 Quantification of ERK activity in ERK knockdown cells. (A) ERK activity in SUM149 shERK1 and shERK2 cells was determined by quantification of the fluorescent signal from the Sox-Sub-D-based peptide sensor. Both ERK1 knockdown and ERK2 knockdown correlated with a decrease in kinase activity. (B) Immunoblotting of phosphorylated ERK isoforms corroborated a decrease in enzyme activity with reduced levels of p-ERK. (C) Correlation between kinase activity and ERK expression intensity confirmed that ERK2 knockdown cells had considerably less activity than control or ERK1 knockdown cells. Graphs were generated using GraphPad.

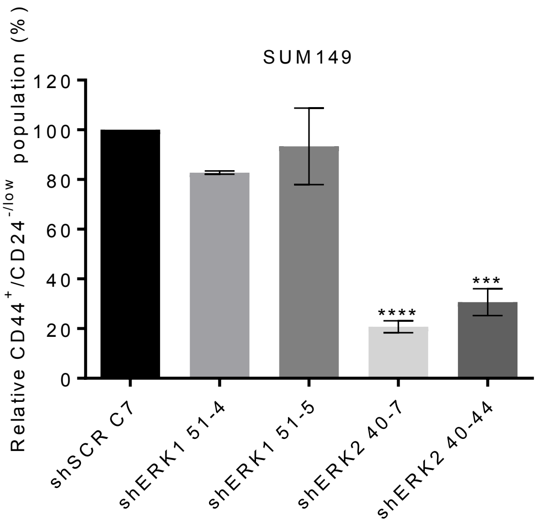

Supplementary Fig.2 Proportion of cells with CD44^{+}/CD24$^{-/low}$ surface marker expression in ERK knockdown cells. SUM149 shERK2 cells had a lower proportion of CD44+/CD24- cells than SUM149 shSCR C7 and SUM149 shERK1 cells. Graphs were generated using GraphPad. Error bars represent the mean (n=3) ± S.D.

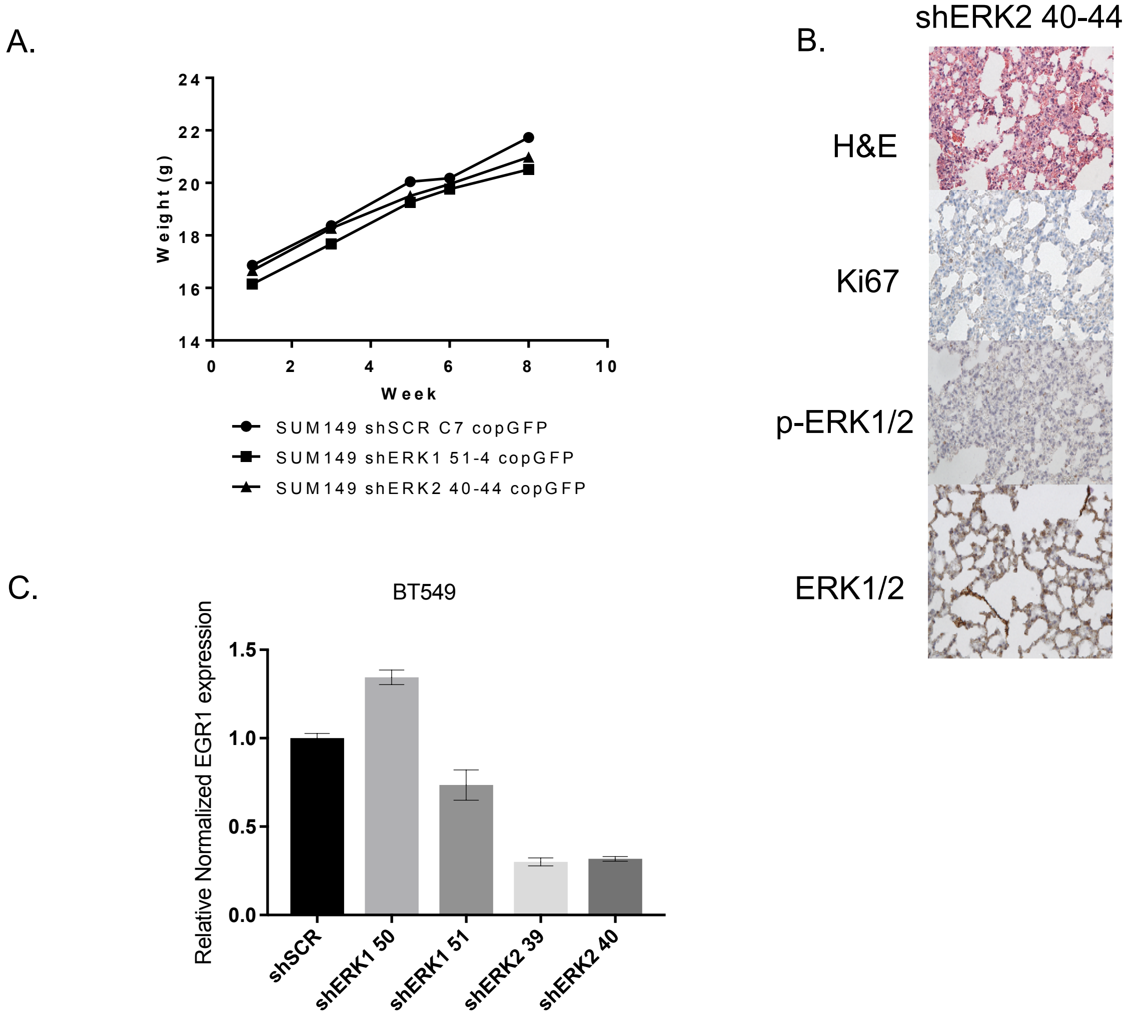

Supplementary Fig.3 Mean weight of mice during SUM149 cell tumor growth and EGR1 expression in ERK knockdown cells. (A) Labeled SUM149 shSCR C7 copGFP, shERK1 51-4 copGFP, and shERK2 40-44 copGFP cells were sorted via flow cytometry and injected into female SCID-beige mice. Weight was monitored every 2 weeks for 8 weeks. (B) Micrographs (20X) of lung SUM149 shERK2 tumors immunohistochemically stained for proliferation marker, ERK, and phosphorylated ERK. (C) qPCR shows that EGR1 expression is significantly reduced in shERK2 39 and shERK2 40 BT549 cells.

Group	N	Events	Median survival (days)	0.95 LCL	0.95UCL	P value
All	45	17	NA	194	NA	
shSCR C7 Control	10	7	183	118	NA	
shERK1	15	9	152	105	NA	
shERK2	20	1	NA	NA	NA	<0.001

Supplementary Table 1. Median overall survival by cell line group and two-sided log rank test results Mice injected with shERK2 cells had significantly better OS than mice injected with SCR C7 cells or shERK1 cells

FDR	Number significant genes	p-value cut-off
0.001	3	1.01e-07
0.005	50	5.80e-06
0.01	147	3.32e-05
0.05	1306	1.90e-03
0.1	3539	1.09e-02

Supplementary Table 2. Number of genes with significant differences in expression between cell line groups. Summary of analyzed Microarray results.

Fig 2 A - Full Blots

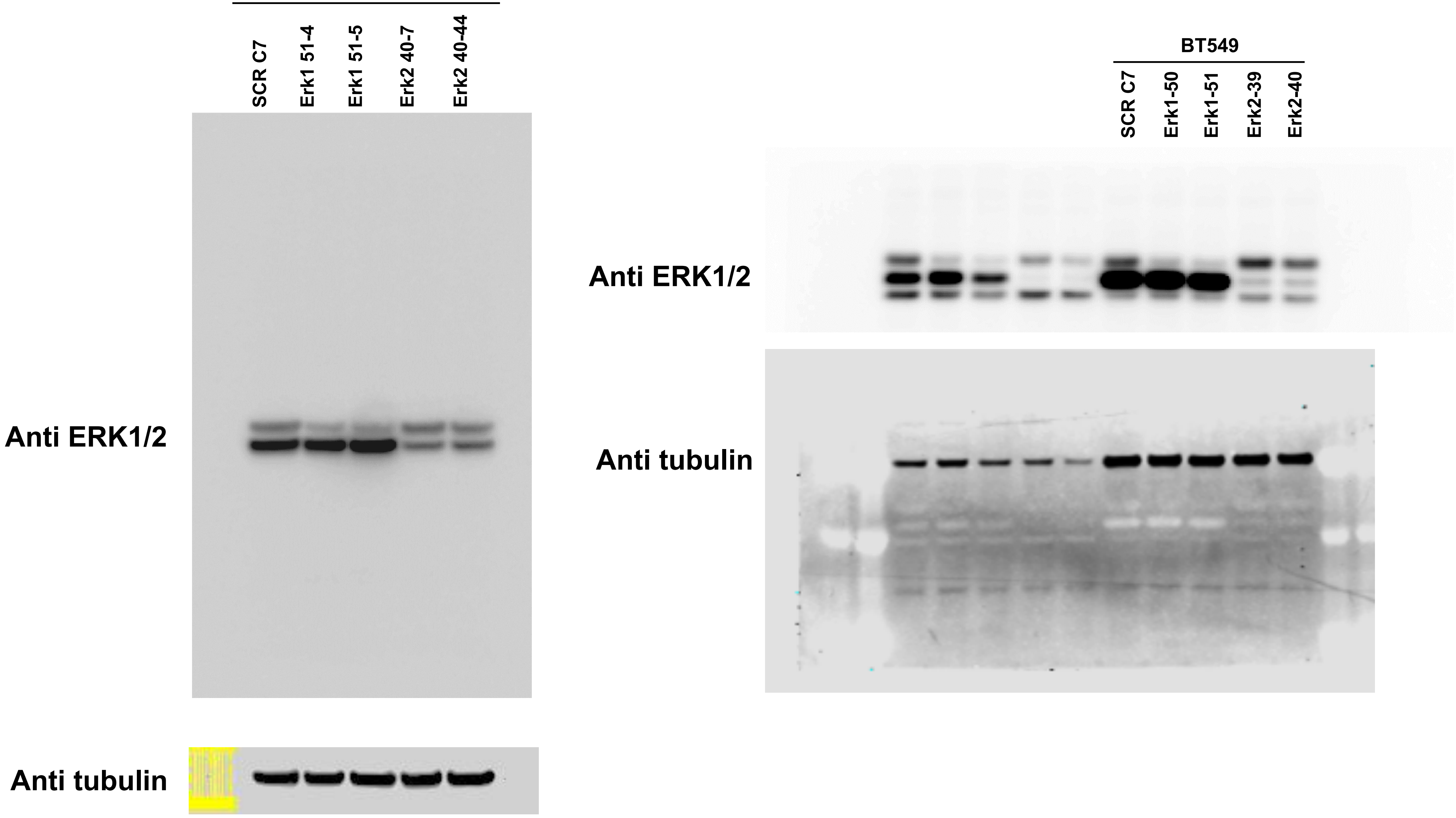

Fig 4A - Full Blots

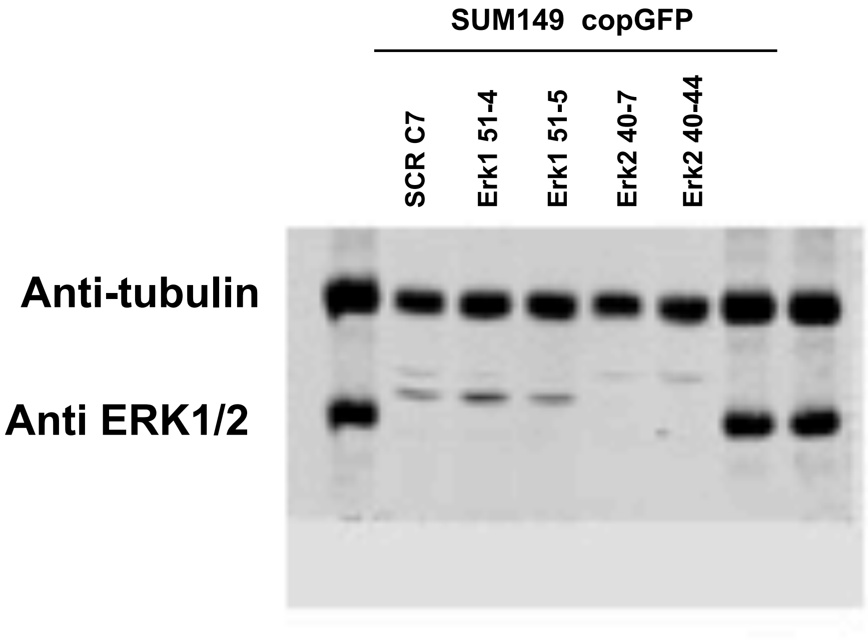

Supplementary Fig 1 - Full Blots

BT549_totalERK_exposure1.tif

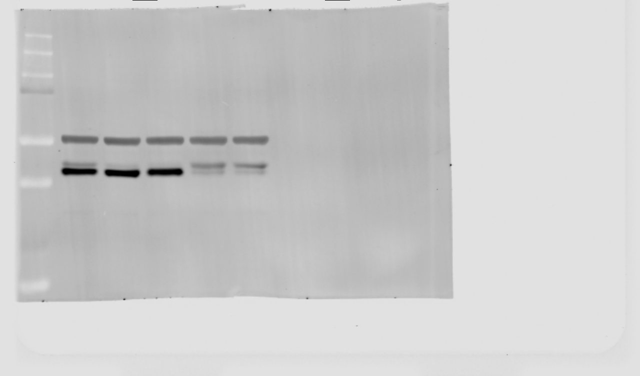

BT549_αTubulin_exposure 2tif

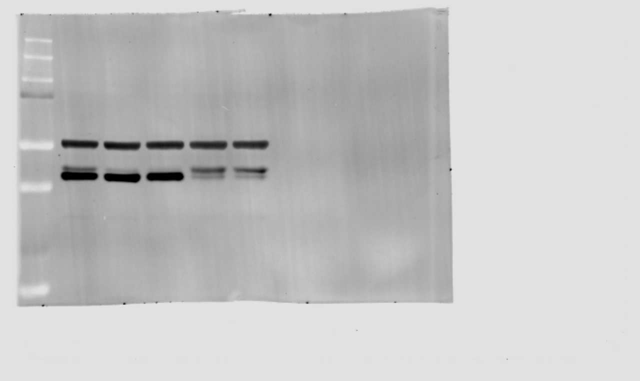

BT549_ppERK.tif

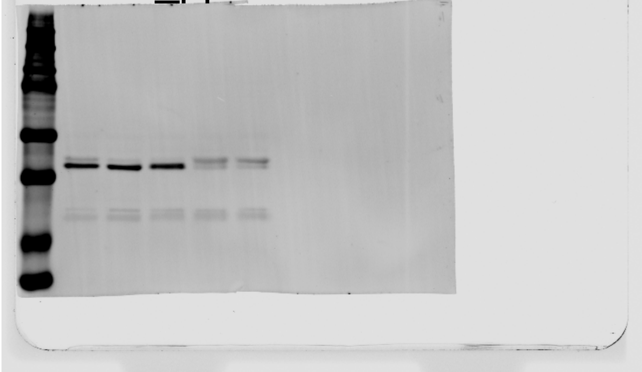

SUM149_ppERK.tif

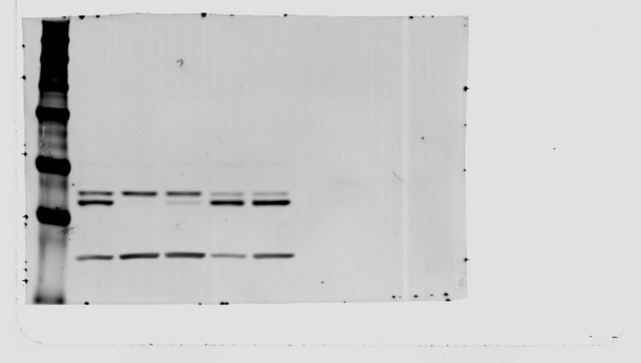

SUM149_totalERK_αTub.tif

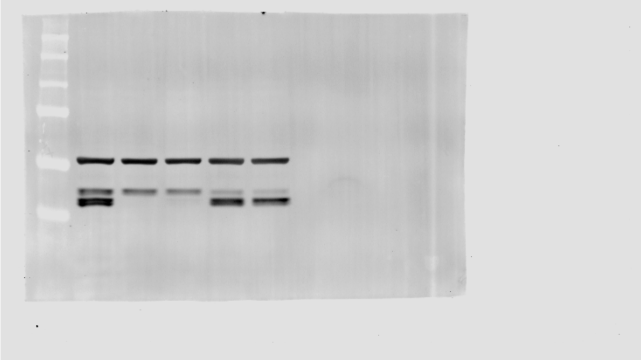

Supplement: Supplementary file 1 — Supplementary Information [file 41598_2020_65250_MOESM1_ESM.pdf]
